# Duplication at 19q13.32q13.33 Segregating with Neuropsychiatric Phenotype in a Three-Generation Family: Towards the Definition of a Critical Region

**DOI:** 10.3390/genes14122157

**Published:** 2023-11-29

**Authors:** Daniele Guadagnolo, Gioia Mastromoro, Barbara Torres, Enrica Marchionni, Francesca di Palma, Marina Goldoni, Dario Cocciadiferro, Antonio Novelli, Laura Bernardini, Antonio Pizzuti

**Affiliations:** 1 Department of Experimental Medicine, School of Medicine and Dentistry, Sapienza University of Rome, Piazzale Aldo Moro 5, 00185 Rome, Italy; 2 Medical Genetics Unit, Department of Diagnosis, Treatment and Transfusional Medicine Services, Fondazione Casa Sollievo della Sofferenza, Istituto di Ricovero e Cura a Carattere Scientifico, 71013 San Giovanni Rotondo (FG), Italy; 3 Laboratory of Medical Genetics, Translational Cytogenomics Research Unit, Bambino Gesù Children Hospital, Istituto di Ricovero e Cura a Carattere Scientifico, 00165 Rome, Italy

**Keywords:** neurodevelopmental delay, *BICRA*, *KPTN*, copy number gain, 19q13.32q13.33 microduplication, chromosomal microarray analysis

## Abstract

Chromosomal submicroscopic imbalances represent well-known causes of neurodevelopmental disorders. In some cases, these can cause specific autosomal dominant syndromes, with high-to-complete penetrance and de novo occurrence of the variant. In other cases, they result in non-syndromic neurodevelopmental disorders, often acting as moderate-penetrance risk factors, possibly inherited from unaffected parents. We describe a three-generation family with non-syndromic neuropsychiatric features segregating with a novel 19q13.32q13.33 microduplication. The propositus was a 28-month-old male ascertained for psychomotor delay, with no dysmorphic features or malformations. His mother had Attention-Deficit/Hyperactivity Disorder and a learning disability. The maternal uncle had an intellectual disability. Chromosomal microarray analysis identified a 969 kb 19q13.32q13.33 microduplication in the proband. The variant segregated in the mother, the uncle, and the maternal grandmother of the proband, who also presented neuropsychiatric disorders. Fragile-X Syndrome testing was negative. Exome Sequencing did not identify Pathogenic/Likely Pathogenic variants. Imbalances involving 19q13.32 and 19q13.33 are associated with neurodevelopmental delay. A review of the reported microduplications allowed to propose *BICRA* (MIM *605690) and *KPTN* (MIM *615620) as candidates for the neurodevelopmental delay susceptibility in 19q13.32q13.33 copy number gains. The peculiarities of this case are the small extension of the duplication, the three-generation segregation, and the full penetrance of the phenotype.

## 1. Introduction

Chromosomal Copy Number Variations (CNVs), like microdeletions and microduplications, can be associated with a variety of neurodevelopmental phenotypes [1]. For many CNVs, the clinical significance has yet to be defined, and most occur in dosage-tolerant regions of the genome [1]. Some are instead associated with specific syndromic phenotypes, usually featuring intellectual disability and dysmorphic features. Commonly, the phenotype is transmitted in an autosomal dominant fashion, and the occurrence of the CNV is often de novo [1]. A third class of recurrent CNVs is usually associated with non-syndromic neuropsychiatric phenotypes with variable penetrance and expressivity [2]. This class of CNVs is mainly investigated, in clinical practice and research, in non-syndromic intellectual disability and autism spectrum disorder cases. They usually result in a wide spectrum of phenotypes, even within the same family [2]. Ascertaining the pathogenicity of CNVs is often more challenging for microduplications than it is for microdeletions, as the human genome is more tolerant towards copy number gains than copy number losses, and bioinformatics tools estimating gene dosage tolerance perform better at identifying haploinsufficiency-susceptible genes rather than copy number gain sensitive ones [3].

We report on a family with non-syndromic neuropsychiatric features segregating across three generations with a novel 19q13.32q13.33 microduplication.

## 2. Case Description

**Figure 1:**
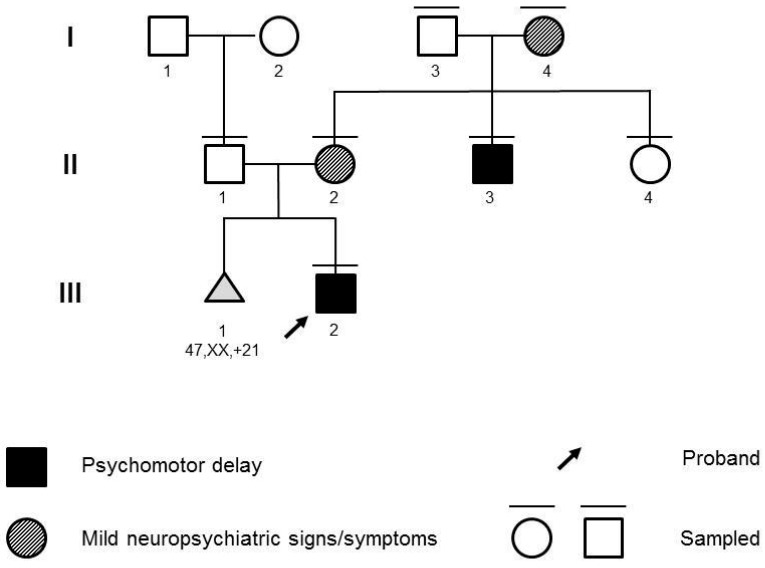
Pedigree of the family. Roman numbers (I, II, III) define generations within the family. Arabic numbers (1, 2, 3, 4) define individuals within each generation.

The propositus (Figure 1; III:1) was a 28-month-old male infant referred for psychomotor delay. He was born at full term of an uneventful pregnancy. Standard fetal karyotype, performed at amniocentesis for a previous miscarriage due to trisomy 21, was normal. Upon clinical examination, mild motor delay and marked speech delay were noted. He started standing and taking steps, with support, at 18 months of age. At 24 months, he started walking on flat surfaces for small distances without support. He started pronouncing the first words by 24 months of age. Upon initial examination, a vocabulary of 5–10 words was reported. He was able to understand short, simple requests and sentences. He presented strabismus. The proband did not present notable facial dysmorphisms or malformations. Abdominal ultrasound and echocardiography scans were normal. Electroencephalograms were also normal. At familial history assessment, the father (Figure 1; II:1) reported a diagnosis of ichthyosis vulgaris. The mother (Figure 1; II:2) reported a brother (Figure 1; II:3) affected by mild intellectual disability, behavior anomalies, fine motor and speech impairment, and a healthy sister (Figure 1; II:4). The mother herself showed signs of anxiety. Upon further investigation, she provided previous personal clinical records reporting Attention-Deficit and Hyperactivity Disorder (ADHD) and learning disability, requiring support teaching.

Chromosomal Microarray (CMA) with SNP-array and Fragile-X Syndrome molecular testing analysis were requested on the proband, and parental samples were collected for potential segregation. CMA was performed with the Affymetrix Cytoscan™ HD platform (Thermo Fisher Scientific, Waltham, MA, USA) and analyzed with Chromosome Analysis Suite v4.0 (Thermo Fisher Scientific, Waltham, MA, USA). Validation and segregation were performed with FISH (N\0026M11 and N0152C07 probes). CMA identified the novel 969 kb 19q13.32q13.33 duplication, arr[GRCh37] 19q13.32q13.33(47765966_48735450)×3. The microduplication was inherited from the mother. The duplication was not reported in literature or databases, but 19q13.32 and 19q13.33 imbalances have been associated with neurodevelopmental phenotypes [4,5]. The rearrangement was uploaded on Decipher with patient ID 412027 (https://www.deciphergenomics.org/patient/412027, last accessed 30 Augus 2023). Fragile-X testing was normal. The family was counseled on the possible contribution of the microduplication to the neurodevelopmental phenotype of the proband and to the psychiatric features presented by his mother (II:2) carrying the CNV. Genetic counseling was offered to all first-degree relatives of II:2 (II:3, II:4, I:3, I:4) to propose segregation analysis. Clinical assessment of II:3 confirmed the reported clinical picture, with intellectual disability, behavioral anomalies, and speech and motor impairment. Strabismus was noted. No other relevant anomalies were identified at clinical examination or retrieved from previous medical records. Personal history and clinical evaluation were not remarkable for II:4 and I:3. The proband’s grandmother I:4, despite not having a formal psychiatric diagnosis, reported phobias and a clinical picture consistent with generalized anxiety disorder and a story highly suggestive of ADHD. Segregation analysis demonstrated the presence of the microduplication in II:3, the affected uncle of the proband, and showed the duplication to be inherited from I:4, also affected. Unaffected individuals (I:3, II:4) did not carry the CNV. To rule out the presence of a monogenic condition, *trio* Clinical Exome Sequencing was performed on proband III:1 and his parents II:1 and II:2, with the Twist Custom Panel (Twist Bioscience, South San Francisco, CA, USA) on the NovaSeq6000 platform (Illumina, San Diego, CA, USA). Read alignment and variant calling were performed with the DRAGEN germline Pipeline (Illumina, San Diego, CA, USA). Variant analysis was performed with the Geneyx Analysis tool (https://geneyx.com/, last accessed on 22 May 2022). No Pathogenic or Likely Pathogenic variants were found. Two heterozygous variants of uncertain significance, both inherited from the unaffected father, were identified in genes implied in neurodevelopmental disorders with autosomal recessive inheritance: the c.3023C>T, p.(Ala1080Val) in *KDM5B* (NM_006618.5), rs575015025 with allele frequency 0.0001591 in GnomAD v2.1.1, and the c.7902_7904delinsAAG, p.(Ser2634Arg) in *HERC2* (NM_004667.6), with no reported allele frequency in GnomAD v2.1.1. *KDM5B*, which is associated with intellectual developmental disorder, autosomal recessive 65 (MIM #618109). *HERC2* is associated with intellectual developmental disorder, autosomal recessive 38 (MIM #615516). Given the severity and inheritance mode of these phenotypes and the heterozygosity of the variants in the proband (with no CNV identified by CMA in the chromosomal regions of these genes), the disorders associated with *KDM5B* and *HERC2* appeared unlikely in the present case, and the role of these variants appeared negligible.

As a result, Clinical Exome Sequencing did not identify causative variants for neurodevelopmental disorders, whereas the 19q13.32q13.33 microduplication segregated with neuropsychiatric features in four individuals across three generations of the family. All individuals harboring the duplication (I:4, II:2, II:3, III:2) presented with neurodevelopmental or psychiatric features, which were absent in non-carriers (I:3, II:4).

## 3. Discussion

We report a novel 19q13.32q13.33 duplication segregating with neurodevelopmental and neuropsychiatric phenotype in a three-generation family. The chromosomal regions 19q13.32 and 19q13.33 are known to harbor copy number gains associated with neuropsychiatric features and usually appear de novo [4,5]. The peculiarities of this case are the chromosomal region involved, the occurrence of three-generation segregation, and the full penetrance of the phenotype, despite broadly variable expressivity.

The duplication segregating in the family encompasses seventeen OMIM genes, of which five are classified as morbid: *KPTN* (MIM *615620), *BICRA* (MIM *605690), *CRX* (MIM *602225), *LIG1* (MIM *619774), and *CARD8* (MIM *609051). *CRX* is implied in a form of autosomal dominant Cone Rod Dystrophy (MIM #120970) and in a form of Leber Congenital Amaurosis (MIM #613829) following an autosomal dominant or recessive trait of inheritance. *CARD8* appears to be possibly implied in Inflammatory Bowel Disease (MIM #619079), whereas *LIG1* is associated with a recessive form of immunodeficiency (MIM #619774). Biallelic variants in *KPTN* are a known cause for neurodevelopmental phenotypes (mental retardation, autosomal recessive 41 MIM #615637) [6]. Heterozygous variants in *BICRA* were recently associated with Coffin–Siris syndrome 12 (MIM #619325) [7]. Given the related phenotypes, *BICRA* and *KPTN* appear to be the best candidates for the neuropsychiatric phenotype of the family. Concerning the other involved genes, *ZSWIM9* might also play a role. It encodes for a protein with unknown function, but with high expression in the human brain [8], and lies in a proposed critical region for intellectual disability [5]. Triplication sensitivity scores were not available for these genes on ClinGen Gene Dosage Sensitivity classification (last accessed 30 August 2023: www.clinicalgenome.org).

**Figure 2:**
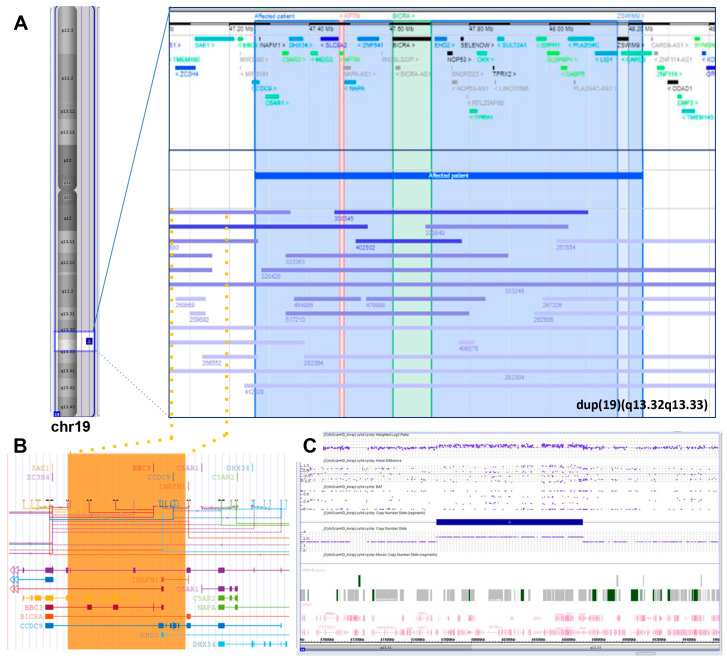
(**A**) The extra copy of the 19q13.32q13.33 region identified by SNP-array in the present case, detailed in (**C**), compared with the other overlapping gains (<3 Mb in size) reported on the DECIPHER database (https://www.deciphergenomics.org/; hg38 release. Last accessed 30 August 2023). Vertical lines highlight the potential candidate genes (*KPTN*, *BICRA*, and *ZSWIM9*) and dotted lines and orange area indicate a fourth candidate area shared by several cases and including a regulatory region of the *BICRA* gene, as reported on the UCSC browser (https://genome.ucsc.edu/ (Last accessed on 30 August 2023); hg38 release) (**B**). The source files for the image are provided in Supplementary Materials Figure S1.

To investigate the role of small copy number gains in 19q13.32q13.33, we searched the DECIPHER v11.22 database (last accessed 30 August 2023) for the GRCh37:19:47765966-48735450 coordinates. The research, excluding the reported case in this study with patient ID 412027, yielded 67 results, with 38 copy number gains. Twenty-one were below 3 Mb in size. When the inheritance was documented, 12/14 gains originated de novo, 1/14 was inherited from a parent with a similar phenotype, and 1/14 originated from a balanced parental rearrangement. This characteristic suggests that this genomic region could be prone to rearrangements and that the clinical consequences of the gains are relevant for neurodevelopment. Nine cases included both *BICRA* and *KPTN*, whereas the 896 kb de novo duplication of patient 256921 featured *KPTN* but not *BICRA*. This patient presented short stature, microcephaly, and intellectual disability, while three cases featured only *BICRA* but not *KPTN* (Figure 2, panel A). Notably, there are three cases spanning the proximal boundary of the present genomic gain that do not include the two most promising genes, but encompass a segment including the known *BICRA* regulatory elements (Figure 2, panel B). Downstream, there are three other cases with no known phenotype that do not include *BICRA* and *KPTN*, but encompass *ZSWIM9* that appears duplicated also in six further larger gains. Based on this search, a possible contribution to the neuropsychiatric might be suggested for *BICRA* and *KPTN*, and possibly *ZSWIM9*.

Literature research for 19q13.32 yielded a single result concerning germ-line copy number gains in the region (PubMed, last accessed 18 August 2023). The results are discussed in the following paragraphs. The studies describing cases with germline 1q13.32 or 19q13.33 copy number gains are presented in Table 1.

Rim et al., reported a patient with microcephaly and developmental delay in 2017 with a de novo 1.3 Mb 19q13.32 duplication [4]. The phenotype was consistent with that of bigger duplications in the same region, albeit less severe, as most of the reported duplications had an extension exceeding 10 Mb. The duplication included both *KPTN* and *BICRA*, further suggesting a possible pathogenic role of their copy-number gains.

The search for 19q13.33 microduplications yielded five results. A paper described a duplication distal to the one we report, in a patient with autism spectrum disorder [9]. One described a large terminal 19q13.33qter duplication [10]. One discussed a case with a severe phenotype and a monosomy 19pter and trisomy 19q13qter resulting from a parental pericentric inversion [11]. A fourth paper described the role of somatic 19q13.33 duplications identified in samples from epileptic patients undergoing brain surgery [12]. A final paper, analyzing cases retrieved from the ClinVar and DECIPHER databases, discussed the role of 19q13.33 duplications in developmental delays, identifying a common region encompassing *CARD8*, *C19orf68* (*ZSWIM9*), *KDELR1*, and *GRIN2D* in children with intellectual disability and suggesting *GRIN2D* as the main candidate for the neurodevelopmental phenotype [5]. Of these, only *CARD8* and *ZSWIM9* are encompassed in the duplication we report.

We then performed further research investigating whether Genome-Wide Association Studies (GWAS) reported the association between Single Nucleotide Polymorphisms (SNPs) in the investigated region and neurodevelopmental phenotypes. We used the GWAS Catalog database (last accessed 15 November 2023: https://www.ebi.ac.uk/gwas/home). No reported association was found for the candidate genes for the neurodevelopmental phenotype discussed above. A single study reporting the association between a locus at 19q13.33 and neurodevelopmental traits was identified [13]. More specifically, the study identified an association between a haplotype encompassing the rs2303690, rs3936340, rs3815908, and rs2560966 SNPs minor alleles and high intelligence in family *trios* assessed for the presence of a child with ADHD [13]. The *ELSPBP1* gene harbors three of the SNPs (rs2303690 in the coding sequence, and rs3936340 and rs3815908 in intronic sequences), whereas the fourth (rs2560966) lies in the intragenic region between *ELSPBP1* and *CABPC5*. These genes are not known to be associated with monogenic conditions and their copy number gain sensitivity has not been assessed. The GWAS findings strengthen the association between the 1q13.33 chromosomal region and neurodevelopment.

The duplication segregating in the family we report is the smallest 19q13.32q13.33 copy number gain described in the literature to be associated with neuropsychiatric phenotype with sufficient confidence. The absence of Pathogenic or Likely Pathogenic variants at Clinical Exome Sequencing adds further strength to the correlation. A possible contribution of the heterozygous VUSs identified in *KDM5B* and *HERC2* to the phenotype, albeit probably unlikely, cannot be completely excluded. This reported three-generation family provides the opportunity to study the penetrance, expressivity, and clinical spectrum possibly associated with this microduplication, as it appears from the literature search [4] and from the DECIPHER database that copy number gains in this region mostly arise de novo. The absence of syndromic features such as abnormal head shape and circumference, or specific dysmorphisms, also appears to be a peculiarity of this family [4]. This report, along with the literature and database research performed, might help define and narrow down a susceptibility region for neurodevelopmental disorders on 19q13.32. If gene dosage mechanisms are to be implied in the pathogenesis, we believe the *BICRA* and *KPTN* genes to be the best candidates. Still, we are aware that assessing or predicting copy number gain sensitivity is challenging, and that more complex mechanisms might underlie pathogenicity for chromosomal microrearrangements.

From this pedigree, we can infer a high penetrance for non-syndromic neurodevelopmental phenotypes, albeit with variable expressivity. All family members carrying the duplication displayed intellectual disability and/or more subtle behavioral and neuropsychiatric phenotypes, involving attention, hyperactivity, learning, or anxiety.

## 4. Conclusions

In conclusion, the segregation of a relatively small 19q13.32q13.33 duplication across three generations with a spectrum of neurodevelopmental phenotypes provides a unique insight into the pathogenicity of copy number gain in the region. Conversely, to most cases, the imbalance is inherited across three generations, while most appear to arise de novo, allowing the definition of an apparently very high penetrance with variable expressivity. The microduplication is the smallest reported in the literature involving the region, and this aids in the identification of a critical region for neuropsychiatric disorders on 19q. The case also provides useful prompts and information for genetic counseling. The relatively mild behavioral or psychiatric phenotypes displayed by some of the carriers with no overt intellectual disability call for a very careful clinical evaluation of family members when studying neurodevelopmental phenotypes and molecular cytogenetic data.

## Figures and Tables

**Table 1:** Studies describing cases with germline 19q13.32 or 19q13.33 copy number gains.

Ref	Phenotype	Inheritance	Coordinates GRCh37/hg19 Chr19	SIZE	Overlap with Present Case
**[4]**	Microcephaly, Developmental Delay, Facial Dysmorphisms	de novo	51,839,641–52,967,920	1.3 Mb	partial
**[9]**	Autism Spectrum Disorder	de novo	49,562,755–49,635,956	73 Kb	no
**[10]**	Intellectual Disability, Seizures, Facial Dysmorphisms	de novo	48,463,121–59,097,842	10.6 Mb	partial
**[11]**	Microcephaly, Developmental Delay, Growth Delay, Facial Dysmorphysms	de novo (parental balanced inversion)	56,927,013–59,128,983	6.9 Mb	no

## Data Availability

Molecular data are available on the DECIHPHER database, with patient ID 412027 (https://www.deciphergenomics.org/patient/412027, last accessed 30 August 2023). Other data are not publicly available, and will be available upon reasonable request from the corresponding author as the clinical reports contain information that could compromise the privacy of involved individuals.

## Supplementary Materials

The following supporting information can be downloaded at: https://www.mdpi.com/article/10.3390/genes14122157/s1.
