# Supplementary figures and images for: Duplication at 19q13.32q13.33 Segregating with Neuropsychiatric Phenotype in a Three-Generation Family: Towards the Definition of a Critical Region
Source: Genes (Basel). 2023 Nov 29;14(12):2157. doi: 10.3390/genes14122157 (PMC10742575; doi:10.3390/genes14122157)

## Slide 1
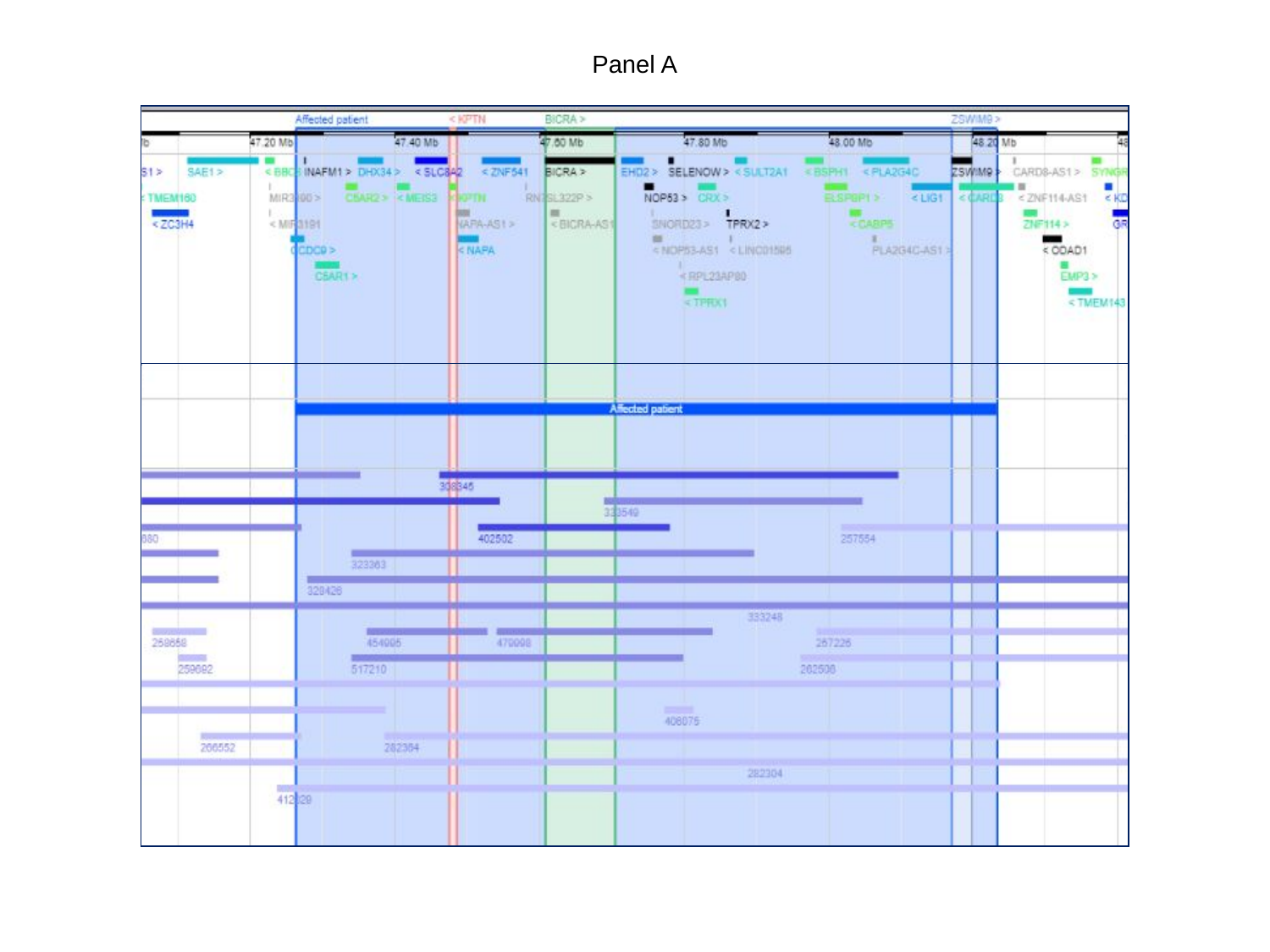

Panel A

## Slide 2
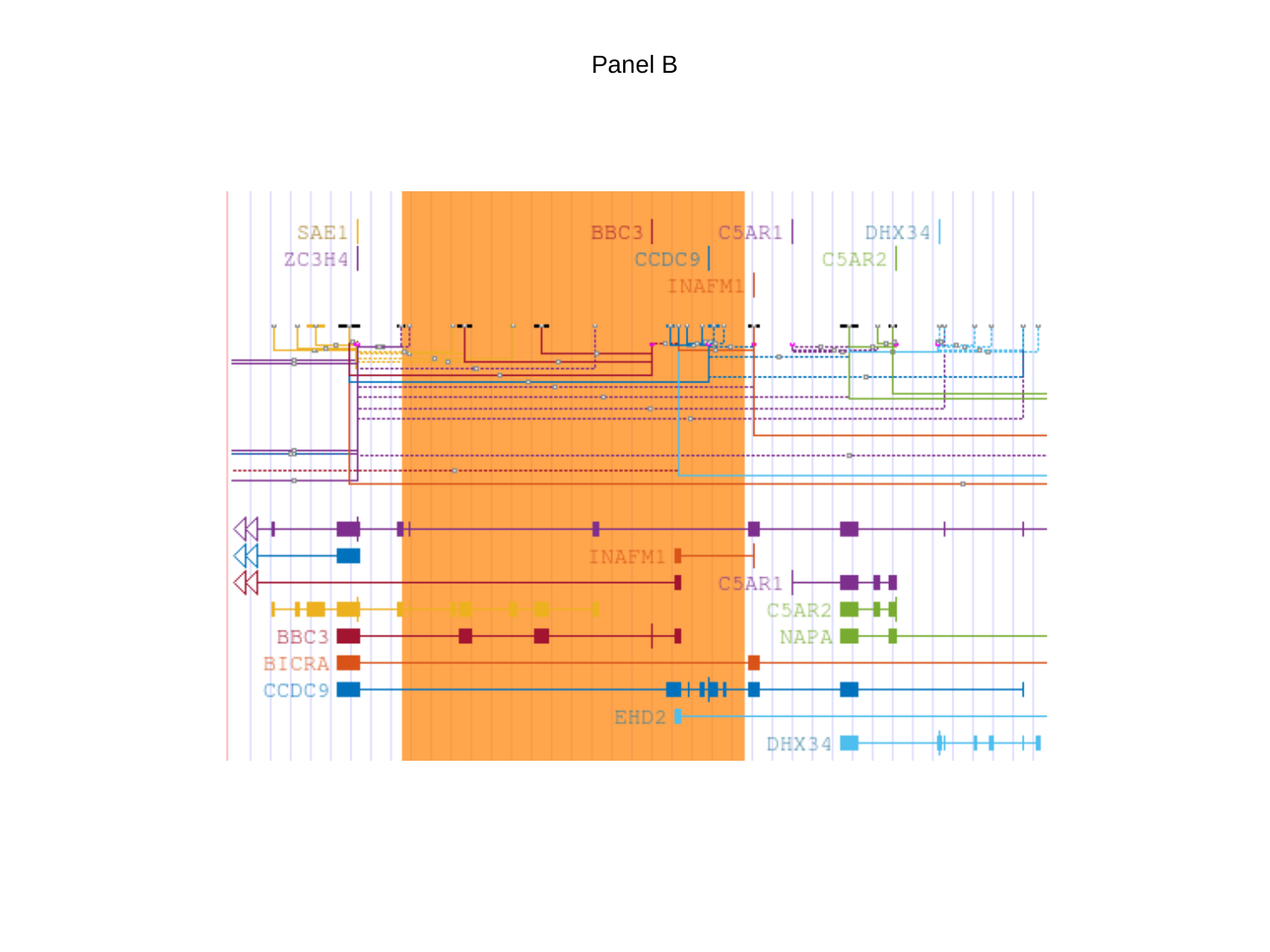

Panel B

## Slide 3
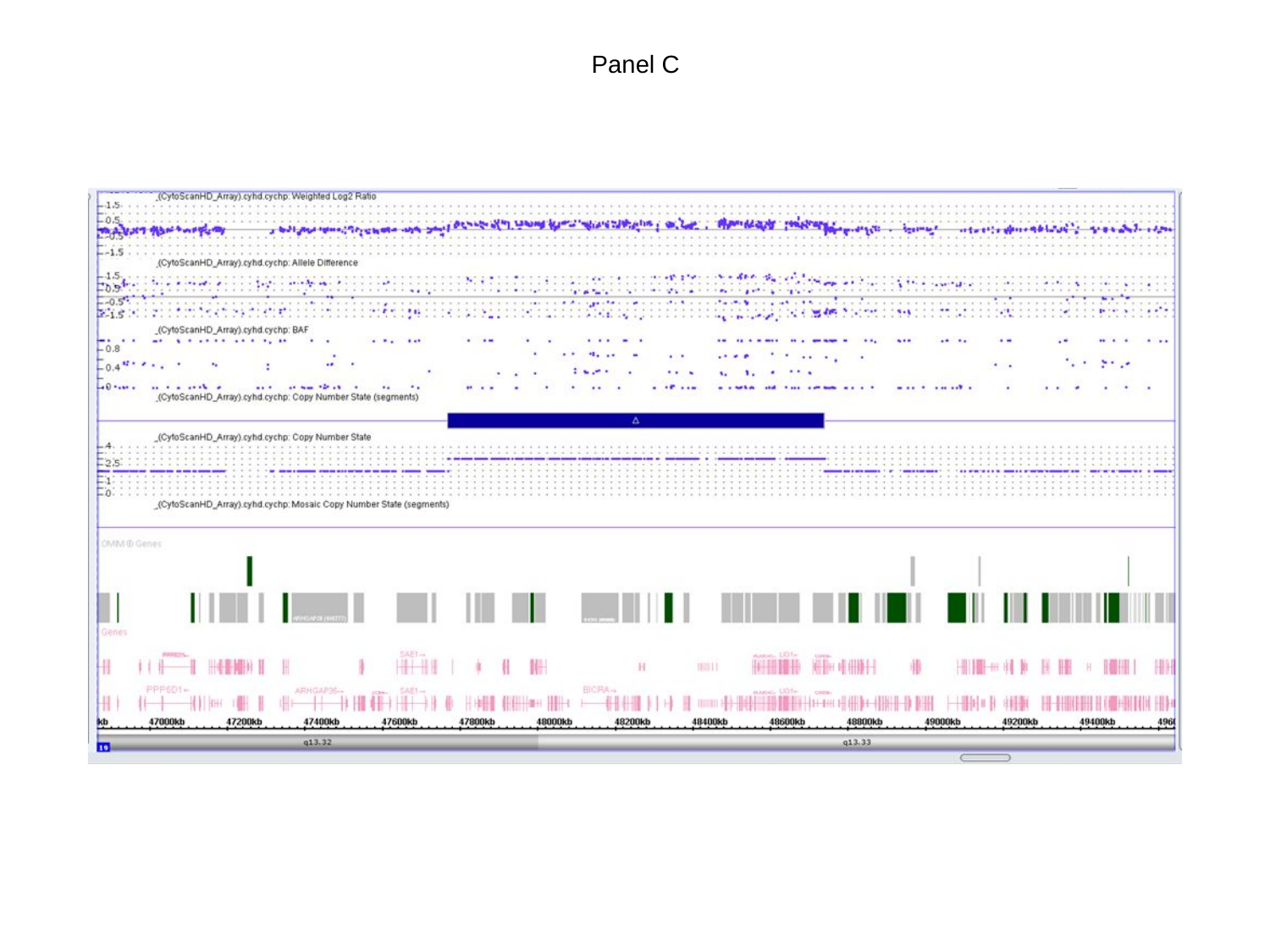

Panel C

Supplement: Supplementary file 1 [file genes-14-02157-s001.zip › genes-2700852-supplementary.pptx]
